# Ultra-low timing jitter, Ti:Al_2_O_3_ synchronization for stimulated Raman scattering and pump-probe microscopy

**DOI:** 10.1117/1.JBO.25.6.066502

**Published:** 2020-06-13

**Authors:** Ben Sherlock, Sarah Saint-Jalm, Graeme P. A. Malcolm, Gareth T. Maker, Julian Moger

**Affiliations:** a University of Exeter, School of Physics and Astronomy, Exeter, United Kingdom; b M Squared Lasers Ltd., Glasgow, United Kingdom

**Keywords:** femtosecond multiphoton microscopy, optical synchronization, pump-probe microscopy, stimulated Raman scattering

## Abstract

**Significance:** Stimulated Raman scattering (SRS) and pump-probe microscopy are implementations of multiphoton microscopy that acquire high-resolution, label-free images of live samples encoded with molecular contrast. Most commercial multiphoton microscopes cannot access these techniques since they require sample illumination by two temporally synchronized ultrafast pulse trains. We present a compact and robust way of synchronizing an additional Ti:sapphire laser with a conventional single-beam multiphoton microscope to realize an instrument that can acquire images with enhanced molecular specificity.

**Aim:** A passive optical synchronization scheme for a pair of commercially available, unmodified modelocked Ti:sapphire lasers was developed. The suitability of this synchronization scheme for advanced biomedical microscopy was investigated.

**Approach:** A pair of modelocked Ti:sapphire lasers were aligned in master–slave configuration. Five percent of the master laser output was used to seed the modelocking in the slave laser cavity. The timing jitter of the master and slave pulse trains was characterized using an optical autocorrelator. The synchronized output of both lasers was coupled into a laser scanning microscope and used to acquire spectral focusing SRS and pump-probe microscopy images from biological and nonbiological samples.

**Results:** A timing jitter between the modelocked pulse trains of 0.74 fs was recorded. Spectral focusing SRS allowed spectral discrimination of polystyrene and polymethyl methacrylate beads. Pump-probe microscopy was used to record excited state lifetime curves from hemoglobin in intact red blood cells.

**Conclusion:** Our work demonstrates a simple and robust method of upgrading single-beam multiphoton microscopes with an additional ultrafast laser. The resulting dual-beam instrument can be used to acquire label-free images of sample structure and composition with high biochemical specificity.

## 1 Introduction

Since its development in 1990, multiphoton microscopy^1^ has become a key enabling technology in the biomedical sciences.^2^^–^^4^ Multiphoton microscopy uses the near-infrared, pulsed output of an ultrafast laser system to excite and image nonlinear optical processes in biological samples. Modern multiphoton microscopes routinely perform live cell imaging at depths >1  mm below the sample surface, with minimal phototoxicity and photodamage.

The majority of commercial multiphoton microscopes use a single ultrafast laser to excite processes such as two photon excited fluorescence or second harmonic generation (SHG) to image sample structure and to a limited degree, composition. However, alternative nonlinear optical processes that require two spatially and temporally overlapped ultrafast pulse trains can be used to provide biochemically specific contrast in nonfluorescent samples. Stimulated Raman scattering (SRS)^5^ and pump-probe microscopy^6^ are examples of dual-beam imaging techniques that probe the intrinsic chemical properties of biomolecules. In SRS, the frequency difference between the two pulse trains is used to excite vibrational resonances in the sample. The chemical specificity of SRS can be further enhanced using spectral focusing, where the relative time delay between two chirped pulse trains is scanned, allowing the acquisition of spectroscopic images.^7^ Pump-probe microscopy records the transient evolution of ground and first electronic excited state populations to provide distinctive signatures from molecules with similar linear absorption spectra.

Both SRS and pump-probe microscopy require precise control of the timing delay between the two pulse trains, and in particular a low pulse-to-pulse timing error, often referred to as timing jitter. Timing jitter on the order of the pulse duration has a deleterious effect on SRS and pump-probe contrast. Low timing jitter between ultrafast pulse trains can be achieved using frequency conversion devices, such as an optical parametric oscillator (OPO) that converts the pulsed input into two tightly synchronized, wavelength tunable output pulse trains. OPOs are widely used tools in biophotonics research laboratories that provide access to a broad range of wavelengths. However, many oscillators used for multiphoton microscopy cannot be easily combined with an OPO. This is because they have either insufficient power, or without using intracavity SHG which increases the burden on the pump power requirement due to conversion inefficiency, the resulting signal/idler tuning range is too far toward the IR. Thus in many cases, integrating an OPO into an existing single-beam multiphoton microscope would require the purchase of an OPO and the replacement of the original oscillator with one that is better suited to pumping an OPO. Turnkey, dual-wavelength synchronized ultrafast laser systems are now commercially available; however, the high cost of these systems represents a significant barrier to their broad adoption.

Integrating a second, tightly synchronized ultrafast laser into a single-beam multiphoton microscope is a cost-effective alternative as it works with, rather than replaces existing instrumentation. Active electronic cavity synchronization schemes have successfully realized few or subfemtosecond timing jitter between the pulse trains of two modelocked lasers.^8^^,^^9^ However, these approaches required the development of high-speed electronic servo loops that dramatically increase the complexity of the laser system. Passive optical synchronization schemes have achieved subfemtosecond timing jitter between the pulse trains of independent cavity-based ultrafast lasers.^10^^,^^11^ However, these approaches required custom-built laser cavities to accommodate a shared Kerr medium. Despite their elegance, the complexity of these approaches means that they are not well suited to adoption by the biomedical research community. In this paper, we demonstrate a simple, passive optical synchronization scheme using unmodified, off-the-shelf commercial ultrafast lasers to realize subfemtosecond timing jitter between the pulse trains. Our approach that involves injecting a fraction of the master laser pulse train into the slave laser cavity has been used previously to synchronize the outputs of solid-state and fiber lasers.^12^^–^^14^ This flexible technique is robust, stable, and independent of the wavelength and polarization state of either laser. In this paper, we demonstrate that our passive optical cavity locking scheme is well suited to femtosecond imaging techniques such as spectral focusing SRS and pump-probe microscopy. This capability represents a significant development for the biomedical sciences research community to whom these techniques are targeted.

## 2 Methods and Results

**Fig. 1:**
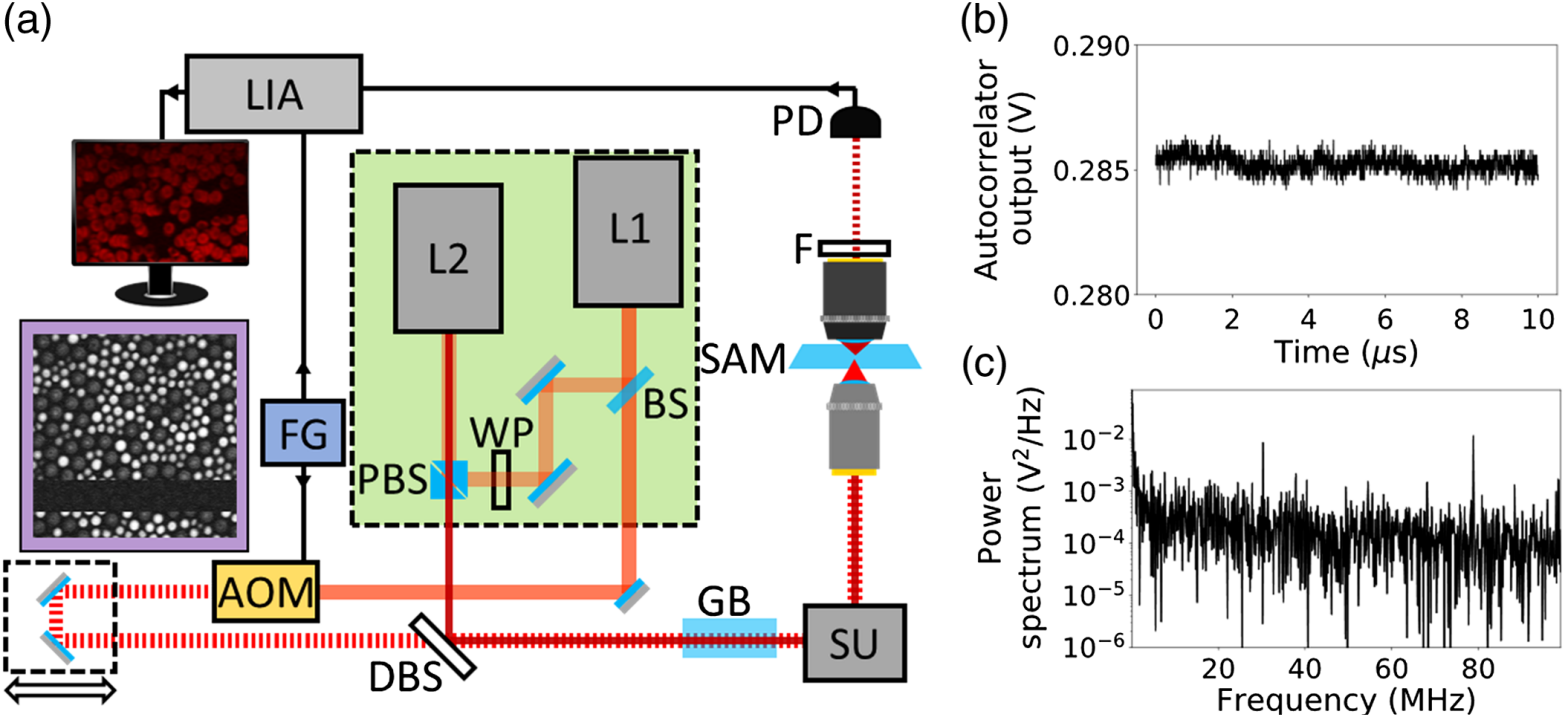
(a) Experimental layout for the optically synchronized dual-beam multiphoton microscope. Synchronization optics shown inside the green rectangle. AOM, acousto-optic modulator; BS, nonpolarizing beam splitter; DBS, dichroic beam splitter; F, spectral filter; FG, function generator; and GB, glass block (only present for spectral focusing stimulated Raman spectroscopy experiments). L1, master femtosecond laser; L2, slave femtosecond laser; LIA, lock-in amplifier; PBS polarizing beamsplitter; PD, photodiode; SAM, sample; and SU, scanning unit. Inset: Stimulated Raman spectroscopy image of PS and PMMA beads. Low-intensity band in the image indicates where synchronization between master and slave lasers was interrupted. (b) Intensity of TPA signal from optical cross correlation of synchronized master and slave beams. Pulses are temporally offset by half a pulse width (half-overlap condition). (c) Power spectrum of optical cross-correlation data showing prominent peaks at 78 and 30 MHz.

A pair of wavelength tunable, modelocked Ti:sapphire lasers (Sprite XT, M Squared Lasers) were aligned in master–slave configuration. The layout of the lasers and optics used for passive synchronization are shown in the green rectangle in Fig. 1(a). The Sprite lasers possess highly stable cavities, and piezo actuated cavity mirrors which make them well suited to passive optical synchronization. A nonpolarizing beamsplitter was used to pickoff 5% of the master output, which is injected into the slave cavity via a pair of mirrors, half waveplate and polarizing beamsplitter cube. The master pickoff is used to seed the Kerr-lens modelocking inside the slave cavity. When the slave cavity repetition rate is matched to that of the master, the slave will lock to the signal from the master, pulsing in tight synchronicity with the master. Provided the average power of the master beam at the input to the slave was >100  mW, we observed robust and stable synchronization at any combination of master and slave wavelengths. We characterized the timing jitter of the synchronized pulse trains using a method based on optical cross correlation that has been used by others in this field.^15^^–^^17^ We recorded the intensity of two-photon absorption (TPA) that resulted when both pulse trains were spatially overlapped and coupled into a gallium phosphide photodiode-based optical autocorrelator.^18^ First, the scanning delay line in the autocorrelator was used to record the optical cross-correlation curve when the pulses trains are maximally temporally overlapped. The linear regions of this curve are used to convert intensity fluctuations to timing fluctuations in the following analysis. Then, with the autocorrelator delay line not scanning, the TPA signal was recorded under two conditions: the master and slave pulse trains were temporally offset by half a pulse width (the half-overlap condition), and the temporal offset was larger than the pulse width (the zero-overlap condition). Signals were recorded without filters or averaging for 10  μs, corresponding to the detection of 800 pulses. In Fig. 1(b), we show the TPA signal in the half-overlap condition. The power spectrum of these data is shown in Fig. 1(c) showing peaks around 80 MHz (corresponding to the laser repetition rate) and 30 MHz which we attribute to an artifact from the autocorrelator detection and amplification electronics. To determine the timing jitter, we calculated the standard deviation of the TPA signal for the two conditions and then converted these values from intensity to timing fluctuations using the linear slope of the cross-correlation signal. Finally, we subtracted the zero-overlap measurement from the half-overlap measurement in quadrature, to find a timing jitter of 0.74 fs. This value is significantly less than the typical laser pulse widths (150 fs), suggesting this approach is well suited to applications in dual-beam multiphoton microscopy. The synchronization scheme showed good robustness to external perturbations, routinely being maintained for >30  min. Recovering synchronization was simply and rapidly achieved by adjusting the slave cavity length using the laser control software.

To demonstrate the biomedical imaging applications for our passively synchronized, dual-femtosecond laser system, we integrated it into a custom laser scanning microscope setup configured for SRS and pump-probe microscopy. The full experimental setup is shown in Fig. 1(a). The output of the master was intensity modulated at 1.24 MHz using an acousto-optic modulator (AOMO 3080-122, Gooch & Housego). Following an optical delay line, the master was spatially overlapped with the slave output using a 785-nm long-pass dichroic beamsplitter (Di02-R785-25×36, Semrock). The combined beams were coupled into a laser scanning microscope where they were focused into samples mounted between two #1.5 coverslips using a 60× water immersion objective (UPLANSAPO60×, Olympus). Nonlinear optical processes in the sample caused a modulation transfer from the master to slave beam. A 100× oil immersion objective (UPLSAPO100×, Olympus) was used to collect the transmitted beams. A 750-nm short-pass filter (FES0750, Thorlabs) removed the master, allowing only the slave beam to be detected by a high-speed silicon photodiode (DET36A2, Thorlabs). The filtered photodiode output was demodulated using a lock-in amplifier (SR844, Stanford Research Systems) and digitized using a data acquisition board (PCI 6110, National Instruments). The inset image in Fig. 1(a) shows the SRS signal from polystyrene (PS) and polymethyl methacrylate (PMMA) beads. The horizontal dark band in the image shows a period during the image acquisition where the synchronization between the two lasers was interrupted. In the absence of optical synchronization, small fluctuations in laser repetition rates impact the temporal overlap of the master and slave pulses in the sample plane, causing a deleterious effect on the SRS signal intensity.

**Fig. 2:**
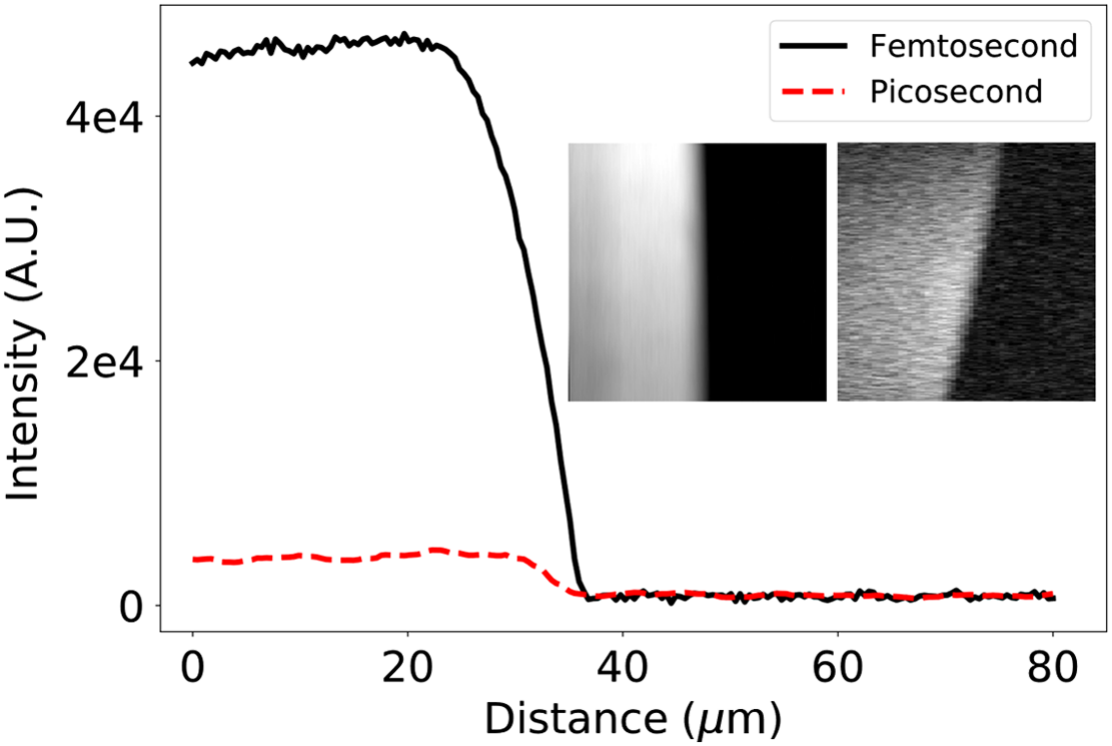
SRS intensity acquired on the C–H stretch bond across an olive oil and air interface using either femtosecond or picosecond pulse trains. Inset (left) femtosecond and (right) picosecond pulsed SRS images of the olive oil to air interface.

To evaluate the quality of our dual-femtosecond laser synchronization scheme, we used SRS images of an olive oil droplet to air interface and compared our results with previously published measurements acquired using a OPO-based femtosecond SRS microscope.^19^ Following^19^ SRS images were acquired using our dual-femtosecond laser system, and an actively synchronized picosecond laser system (Levante Emerald, APE) operating with the same average power. We calculated the SRS signal-to-noise ratio (SNR) for the olive oil to air interface images acquired using femtosecond and picosecond pulses. The ratio of these SNRs provided a means to quantitatively compare the performance of our dual-femtosecond laser system against the same measurement made by other researchers using an OPO-based femtosecond SRS microscope. Both laser systems were tuned to a Raman shift of $2947\;{\mathrm{cm}}^{-1}$ to coherently excite the C–H stretch bond in olive oil. For the femtosecond laser system, we used a 725-nm, 9-mW slave (pump) beam and 922-nm, 6-mW master (Stokes) beam. The picosecond SRS images were acquired using a 798-nm, 9-mW slave (pump) beam, and a 1032-nm, 6-mW master (Stokes) beam. In Fig. 2, we show the intensity of the SRS signal across the olive oil to air boundary acquired using the dual-femtosecond and dual-picosecond laser systems. We calculated a ratio of 11.2 between the mean SNR of the femtosecond to picosecond SRS signals.

**Fig. 3.**
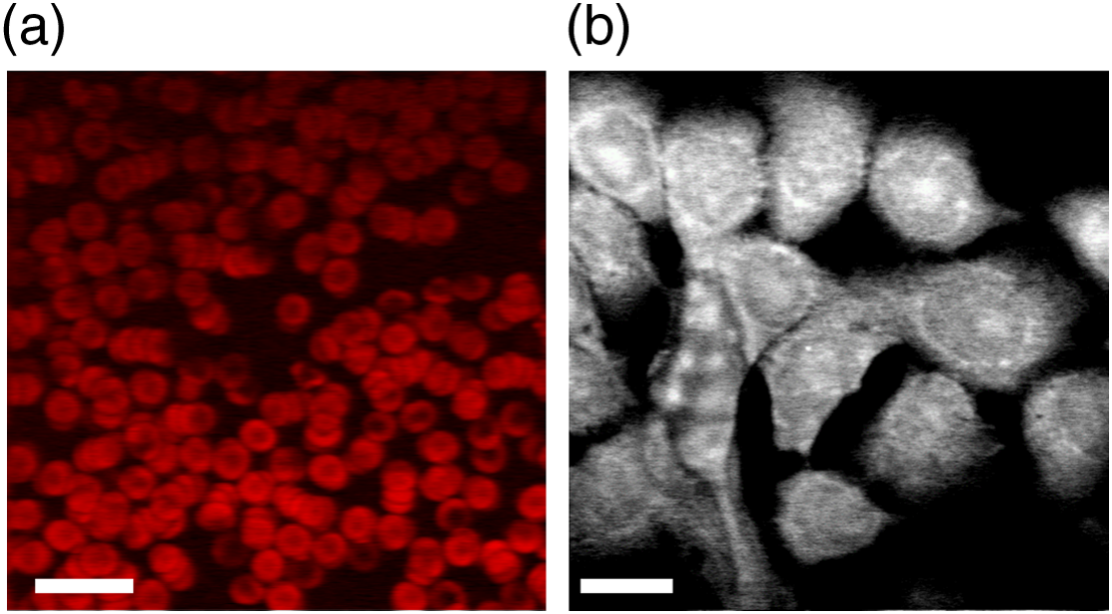
Label-free multiphoton microscopy images of cells. (a) ESA image of hemoglobin in RBCs. Scale bar is 20  μm. (b) SRS image of fixed cancer cells on the $2970\text{-}{\mathrm{cm}}^{-1}$ band. Scale bar is 5  μm.

SRS is commonly used to image the molecular composition in thin samples such as cells. In Fig. 3, we present label-free images of red blood cells (RBCs) and fixed cancer cells acquired using the dual-femtosecond laser system. Contrast in the RBC image derives from excited state absorption (ESA) in hemoglobin using 810-nm, 6-mW master and 740-nm, 2.4-mW slave beams. SRS images of the $2970\text{-}{\mathrm{cm}}^{-1}$ band in fixed cancer cells were acquired using a 924-nm, 12-mW master and 725-nm, 13-mW slave beam. The clarity of these images is a strong indicator that our passively synchronized dual-femtosecond laser system will readily find applications in biomedical research.

**Fig. 4:**
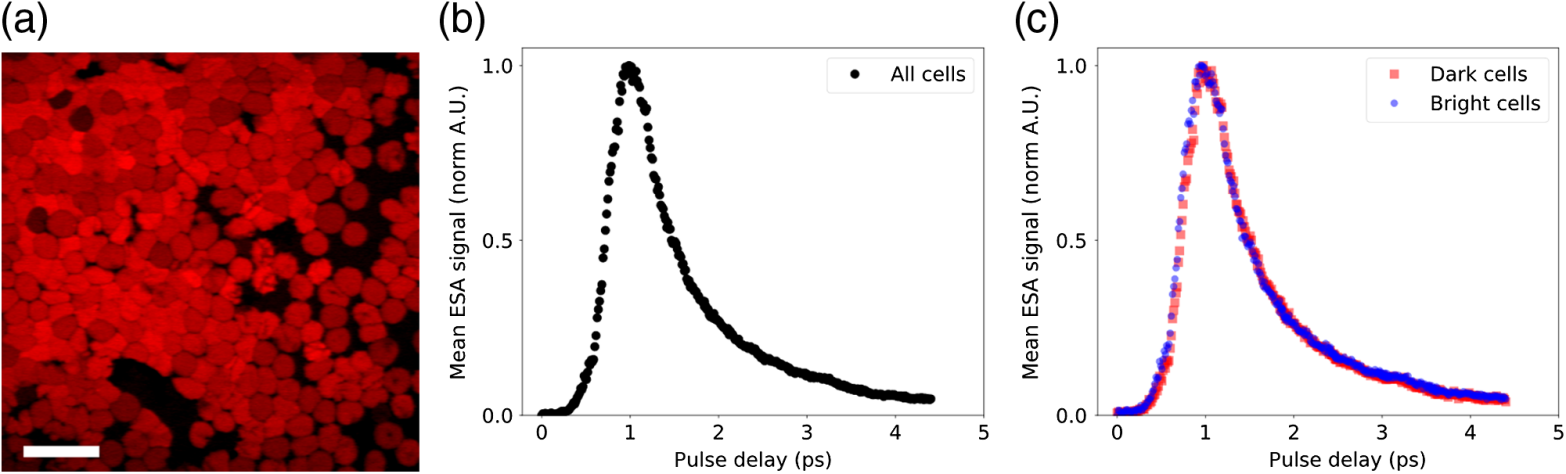
Transient absorption in RBCs. (a) ESA image of coverslip mounted RBCs. Scale bar is 20  μm. (b) Mean normalized ESA signal as a function of pulse delay from all RBCs. (c) Mean normalized ESA signal as a function of pulse delay from five dark RBCs and five bright RBCs.

To further investigate the stability of the passive synchronization scheme, we conducted imaging studies that place stringent demands on timing jitter of the two ultrafast pulse trains. First, we investigated the suitability of our setup for recording the ultrafast dynamics of ESA in the hemoglobin of fresh coverslip mounted RBCs. To acquire the hemoglobin transient absorption curves shown in Fig. 4, we recorded 486 512×512 pixel (pixel dwell time of 8  μs) ESA images of hemoglobin while scanning the optical delay line in steps of 13 fs for each image. Measurements were made using 810-nm, 6-mW master and 740-nm, 2.4-mW slave beams. To provide a qualitative measure of the instrument temporal response function, we also acquired four-wave mixing (FWM) images from titanium dioxide nanoparticles while scanning the optical delay line. A comparison between the temporal response of the ${\mathrm{TiO}}_{2}$ FWM and hemoglobin ESA is shown in Supplemental Fig. S1.

**Fig. 5:**
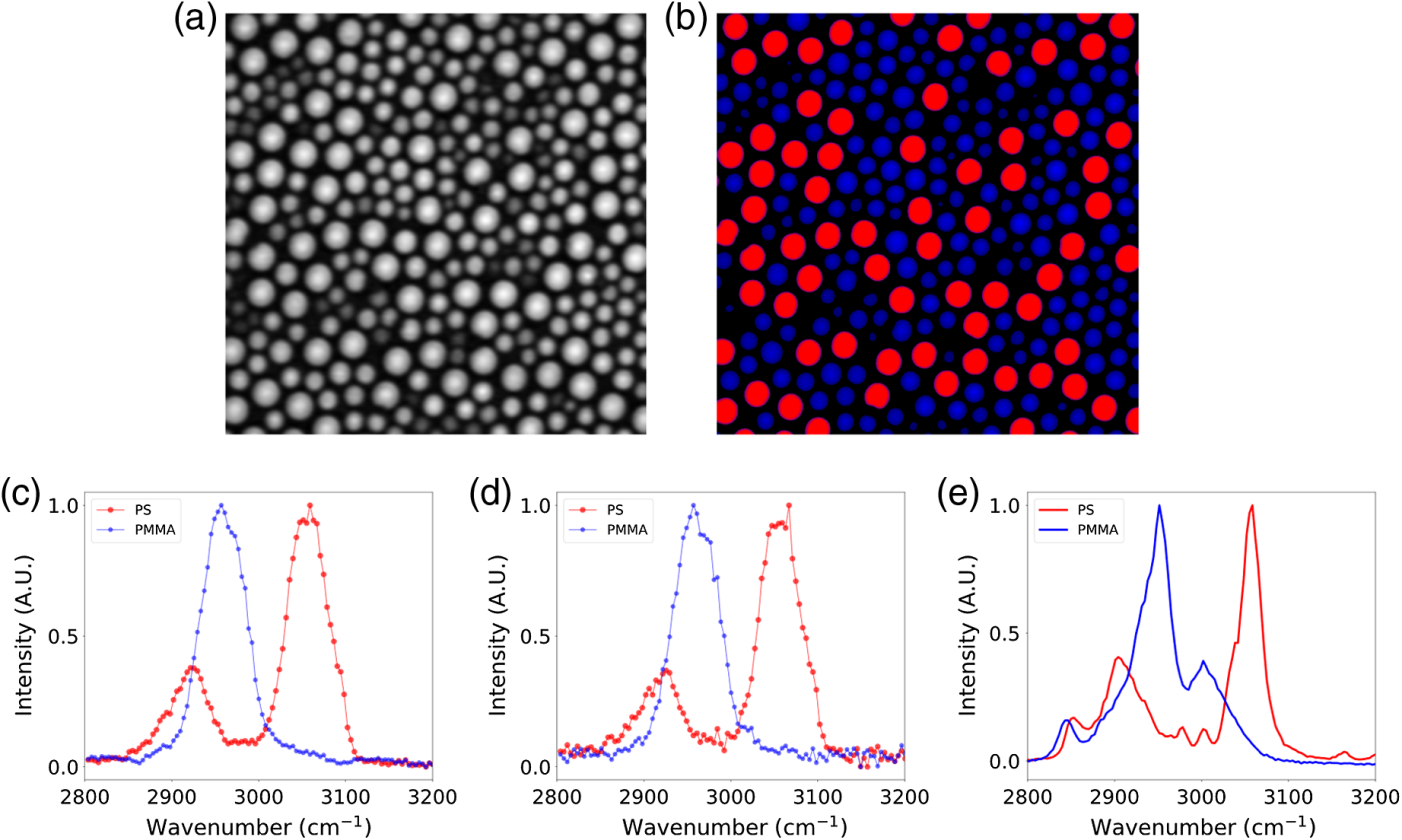
Spectral focusing SRS using mixtures of PS and PMMA beads. (a) SRS image of bead mixture. (b) Color coded images of PS (red) and PMMA (blue) beads based on SRS spectral decomposition. (c) Normalized PS and PMMA spectra acquired by averaging all PS and PMMA beads within the field of view of the hyperspectral image stack. (d) Normalized PS and PMMA spectra acquired by averaging pixels from a single PS (red) and PMMA (blue) bead across the hyperspectral image stack. (e) Normalized spontaneous Raman spectra from PS and PMMA beads.

Finally, we performed spectral focusing SRS in mixtures of PS and PMMA beads. A 35-mm long glass block was introduced into the common path of the master and slave beams to chirp the pulses. Following transmission through the glass block, the full-width at half-maximum of the slave laser at 727 nm had increased to 321 fs, and the master laser at 927 nm to 442 fs. The chirped pulses could now be used for spectral focusing measurements, where an adjustment of the master beam optical delay line resulted in a different vibrational resonance being driven in the sample. In Fig. 5, we show the results of these spectral focusing SRS measurements extracted from 125 images of a mixture of PS and PMMA beads. All images acquired using 927-nm, 6-mW master and 727-nm, 2.4-mW slave beams. Figure 5(a) shows a single SRS image of the sample with limited contrast between bead types. A spectral decomposition of the full spectral focusing SRS image stack allowed straightforward discrimination of bead types [Fig. 5(b)]. The spectra in Fig. 5(c) were found by averaging all the pixels identified as either PS or PMMA beads across an entire image. In Fig. 5(d), we show that these spectra can still be recovered from only a small number of pixels within a single PS or PMMA bead. To provide a qualitative validation of PS and PMMA spectra measured using spectral focusing SRS, Fig. 5(e) shows the spontaneous Raman spectra acquired from the same sample using a commercial Raman confocal microscope (alpha300 R, WITec).

## 3 Discussion

Passive optical synchronization of two (or more) commercial Ti:sapphire lasers presents a comparatively simple and robust approach to enhancing the imaging capabilities of existing multiphoton microscopes. In this work, we investigated the suitability of a dual-femtosecond laser system employing optical synchronization for biomedical imaging applications. We use image-based metrics from SRS and pump-probe microscopy to compare our synchronization technique against more complex and expensive OPO-based microscopes. In Fig. 2, we measured the SNR enhancement of femtosecond over picosecond SRS when driving the C–H stretch bond in an olive oil sample. Our measurement of a factor of 11.2 enhancement agrees with a similar measurement made by Cheng et al.,^19^ who reported an enhancement factor of 12 using an OPO-based system. In Fig. 4, we used ESA to analyze the transient absorption of hemoglobin in fresh, coverslip mounted RBCs. The smooth transient absorption curves show good agreement with those reported by Warren et al.^20^ using an OPO-based system at similar wavelengths and average powers. In these images, we observed heterogeneity in the ESA strength in certain RBCs. To investigate this further, we analyzed the transient absorption curves for the five darkest and five brightest RBCs. Despite the difference in peak ESA, we did not observe any difference in the normalized transient absorption curves. The hemoglobin content of RBCs decreases throughout their lifetime, and given that any blood sample will contain a distribution of RBC ages, we postulate that this may be responsible for the observed heterogeneity. In Fig. 5, we show the partial spectra of PS and PMMA beads measured using spectral focusing SRS. These spectra show good agreement with the spontaneous Raman spectra and previously published SRS spectra for these samples.^7^^,^^21^ We have shown in these final two examples that our passive optical synchronization technique can support imaging techniques that place demanding requirements on the timing jitter between spatially overlapped ultrafast pulse trains.

## 4 Conclusion

In this work, we present a compact, robust, and convenient approach to expand the label-free imaging capabilities of single-laser beam multiphoton microscopes. Our technique based on the passive optical synchronization of two modelocked Ti:sapphire lasers operates independently of the wavelength and polarization state of both lasers. We have shown that this approach realizes subfemtosecond timing jitter between two ultrafast pulse trains which can be used to excite a variety of nonlinear optical processes across the Ti:sapphire tuning range. In this work, we used two lasers with piezoadjustable cavity mirrors. However, in principle, this technique only requires one laser to have an electronically adjustable repetition rate which can be matched to the other. In future work, we aim to demonstrate passive optical synchronization of different types of ultrafast laser system and thereby demonstrate how readily this work could be used to upgrade existing single-laser beam multiphoton microscopes. We believe that this work will bolster the burgeoning interest in label-free, biochemically specific microscopy techniques in the biomedical research community.
