# Supplementary material for: Ultra-low timing jitter, Ti:Al2O3 synchronization for stimulated Raman scattering and pump-probe microscopy
Source: J Biomed Opt. 2020 Jun 13;25(6):066502. doi: 10.1117/1.JBO.25.6.066502 (PMC7294598; doi:10.1117/1.JBO.25.6.066502)
Supplement: Supplementary file 1 [file JBO_025_066502_SD001.pdf]

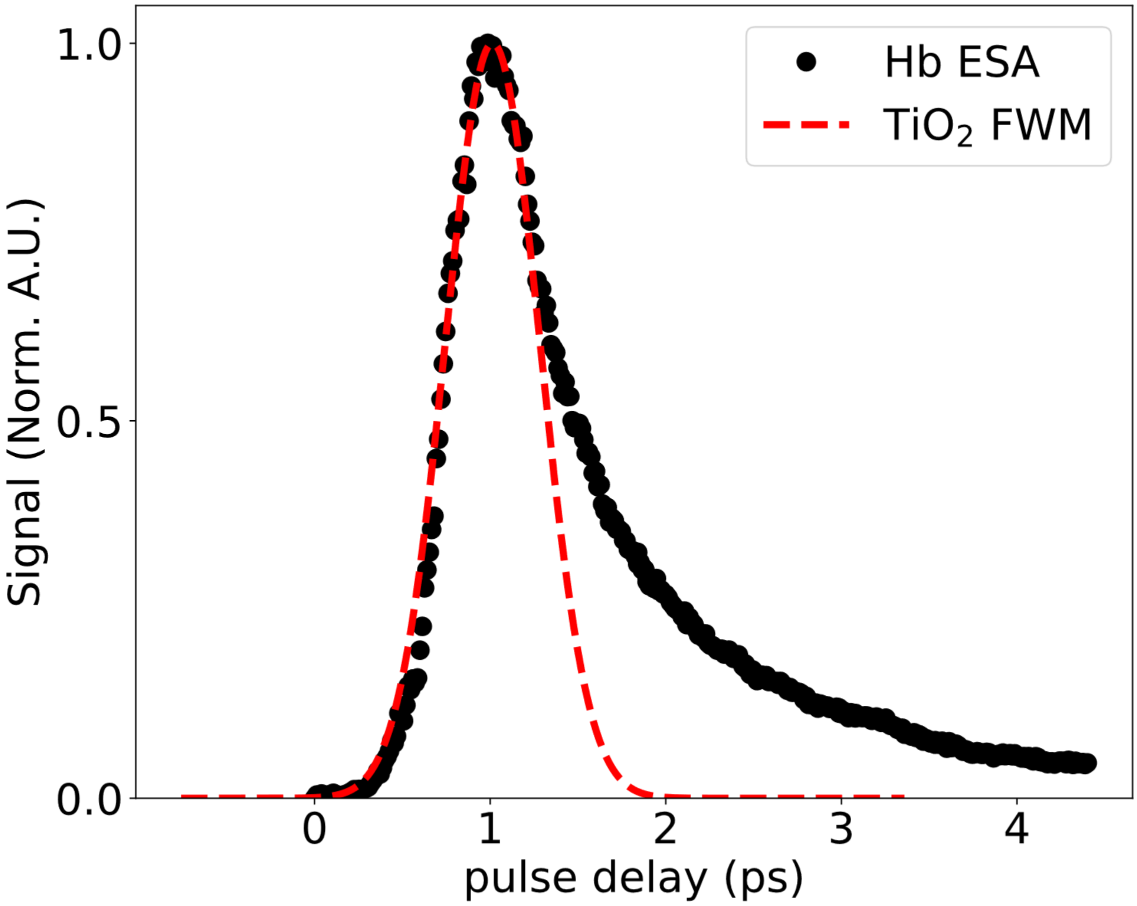

Supplemental Fig. 1 Comparison of temporal dynamics of haemoglobin excited state absorption (ESA) signal (black) with a gaussian fit to the four-wave mixing (FWM) signal from TiO_2 nanoparticles. The ESA data were acquired using 810 nm master and 740 nm slave beams. The FWM data were acquired using 927 nm master and 727 nm slave beams. Furthermore, the FWM data were acquired with chirped pulses due to the introduction of a 35 mm glass block into the common path of the master and slave beams.
